# The *Mycoplasma conjunctivae *genome sequencing, annotation and analysis

**DOI:** 10.1186/1471-2105-10-S6-S7

**Published:** 2009-06-16

**Authors:** Sandra P Calderon-Copete, George Wigger, Christof Wunderlin, Tobias Schmidheini, Joachim Frey, Michael A Quail, Laurent Falquet

**Affiliations:** 1 Swiss Institute of Bioinformatics, Génopode-UNIL, 1015 Lausanne, Switzerland; 2 Microsynth AG, Schützenstrasse 15, P.O. Box, 9436 Balgach, Switzerland; 3 Institute for Veterinary Bacteriology, University of Bern, Länggass-Strasse 122, 3012 Bern, Switzerland; 4 Wellcome Trust Genome Campus, Hinxton, Cambs CB10 1SA, UK

## Abstract

**Background:**

The mollicute *Mycoplasma conjunctivae *is the etiological agent leading to infectious keratoconjunctivitis (IKC) in domestic sheep and wild caprinae. Although this pathogen is relatively benign for domestic animals treated by antibiotics, it can lead wild animals to blindness and death. This is a major cause of death in the protected species in the Alps (e.g., *Capra ibex*, *Rupicapra rupicapra*).

**Methods:**

The genome was sequenced using a combined technique of GS-FLX (454) and Sanger sequencing, and annotated by an automatic pipeline that we designed using several tools interconnected via PERL scripts. The resulting annotations are stored in a MySQL database.

**Results:**

The annotated sequence is deposited in the EMBL database (FM864216) and uploaded into the mollicutes database MolliGen  allowing for comparative genomics.

**Conclusion:**

We show that our automatic pipeline allows for annotating a complete mycoplasma genome and present several examples of analysis in search for biological targets (e.g., pathogenic proteins).

## Background

Mycoplasmas (class Mollicutes) are among the smallest microorganisms capable of self-replication and autonomous life [1]. The genus Mycoplasma includes a large number of highly genomically-reduced species which in nature are associated with hosts either commensally or pathogenically [2]. General features of the class Mollicutes are small genome, lack of cell wall and low GC content.

Indeed, the Mycoplasma species have genomes of 0.6 to 1.3 Mbp. Weisburg et al. (1989) [3] and Woese et al. (1980) [4] revealed that Mycoplasma have evolved from more classical bacteria of the firmicutes taxon by a so-called regressive evolution that resulted in massive genome reduction [5] and minimal metabolic activities. Consequently, they adopted a strict parasitic life style, mainly occurring as extracellular parasites often restricted to a living host, with some species having the ability to invade host cells as described by Sirand-Pugnet et al. (2007) [5], Rosengarten et al. (2000) [6] and Citti et al. (2005) [7]. They have a predilection for the mucosal surfaces, where they successfully compete for nutrients with many other organisms, establishing chronic infections [5]. They do not show specific virulence factor as known in other bacteria, instead they seem to use toxic metabolic intermediates that they secrete and translocate to the host cells as virulence factors [8]. Additionally, due to the lack of cell wall, they are not affected by some antibiotics which target synthesis of cell wall such penicillin or other beta-lactam antibiotics making these organisms particularly interesting in medicine.

### Infectious keratoconjunctivitis (IKC)

*Mycoplasma conjunctivae *is considered as the major etiological agent of Infectious KeratoConjunctivitis (IKC) for both domestic and wild caprinae species. In the European Alps it affects several species such as alpine ibex (*Capra ibex ibex*), alpine chamois (*Rupicapra rupicapra rupicapra*), and mouflon (*Ovis orientalis musimon*), as well as in domestic sheep and goat [9]. In Switzerland, *M. conjunctivae *is known to be the primary cause of this disease [10].

The implied role of *M. conjunctivae *is based on the frequent isolation of this organism from inflamed eyes and on limited attempts to induce ocular disease experimentally showing that *M. conjunctivae *is one agent responsible for epidemic keratoconjunctivitis [11]. Nonetheless, even if the molecular epidemiology has been well described by Belloy *et al. *(2003) [9], the molecular infection mechanism is still not established and remains a mystery.

## Methods

### Bacterial strain

*M. conjunctivae *type strain HRC/581^T ^(NCTC10147) [12] was grown on standard mycoplasma broth medium enriched with 20% horse serum, 2.5% yeast extract and 1% glucose (Axcell Biotechnologies). The cells were harvested by centrifugation at 13,000 × g for 20 min, washed three times in TES buffer (10 mM Tris-HCl, 1 mM EDTA, 0.8% NaCl, pH 7.5), and then re-suspended in TES buffer to a concentration of approximately 109 bacteria/ml. DNA was extracted by the guanidium thiocyanate method [13], extracted 3 times with PCIA (Phenol: CHCl_3_: Isoamylalcohol = 49.5: 49.5: 1) and 3 times with CIA (CHCl_3_: Isoamylalcohol = 99: 1), precipitated with 50% isoproanol, washed 2 times with 70% ethanol to remove salt, dried in the air for 15 min and re-suspended in double distilled H_2_O at a concentration of 500 μg/ml.

### Sequencing

Sequencing and assembly of the genome was carried out by Microsynth AG. The quality of the isolated genomic DNA was verified by gel electrophoresis and displayed a pure high molecular weight DNA. The DNA was sheared by passing it several times through a needle, in order to construct two different libraries: a plasmid library and a fosmid library. For the plasmid library (2–12 Kbp inserts), the genomic DNA was passed 30 times through a 30-Gauge needle and sonicated for 10 seconds (sonication strength 3 on a Digital Sonifier 450 from Branson Ultrasonics corp, Danbury, CT, USA). For the fosmid library (32 Kbp inserts), the genomic DNA was passed 10 times through a 23-Gauge needle without sonication.

Small fragments were ligated with a linker, fractionated twice through 0.8% agarose gels. Fractions of 6 different sizes (from 2 to 12 Kbp) were cut out from the gel and cloned into vector pOTW12 (Sanger Institute). Moreover, the large fragments were fractionated using a CHEF-DR II System (BIORAD). Fragments of 32 Kbp were cut out from the gel and ligated into pCC1Fos (Epicentre Biotechnology Inc.).

From the plasmid library 11'300 clones and from the fosmid library 384 clones were end-sequenced on an ABI 3730 capillary sequencer. A second part of the small fragments were sequenced using 454 Life Science FLX technology leading to 263'163 reads that were reduced to 78'498 reads covering 20'569'079 bp after applying a quality cut-off filter (approx. 22× coverage).

### Assembly

The assembly was carried out using the SeqMan module of the DNASTAR Lasergene version 7 combining both classical Sanger sequences (ABI3730) and 454 FLX reads. A check was conducted with "amosvalidator" of the AMOS package [14], allowing identifying suspicious regions in the assembly. To help in the assembly process the 384 fosmids paired-end reads were aligned to the final sequence. The reads display a nice spreading at regular intervals except for 2 clones that were absent from the results. The 2 regions were analyzed for the presence of potentially lethal genes for *E. coli*. The first region contains a homologue of the gene lepA that is known to be lethal when overexpressed in *E. coli *[15]. This region also contains a restriction enzyme that might cut *E. coli *genome. The second region contains a transposase and some phage genes. This might explain the toxicity of these two fosmids in *E. coli*.

### Automatic annotation

The automatic annotation pipeline was entirely built locally using available software and linking them with Perl scripts.

Gene prediction was carried out using Glimmer 3.02 [16] and the genetic code specific for Mycoplasma (e.g., UGA encodes a tryptophane). The interpolated context models (ICM) were calculated by self-training on the long ORFs of the contigs. The RNAs were predicted using Infernal with models obtained from RFAM [17], tRNAscan-SE [18], and blastn for 16S and 23S [19].

Predicted coding sequences (CDS) were translated using the EMBOSS package (extractseq, transeq, revseq) [20] and a similarity search was run by blastp against the UniProt/Swiss-Prot knowledgebase (Release 56.2 of 23-Sep-2008: 398181 entries) [21]. The CDS were also scanned against the HAMAP families [22] to identify orthologous protein families. In addition the CDS were searched for potential known domains using InterProScan [23], and for biased compositional regions with SEG [24] and Marcoil [25].

The biological interest of an annotation project is to identify the gene products by designating a descriptive common name for the protein and its function with as much specificity as the evidence supports. We use homology-based annotation transfer to assign the name and associated information of gene product: Gene symbol, EC number if protein is identified as an enzyme and other features.

Homology search is performed by blastp that allows finding the best matches with the highest significant sequence similarity appearing between the putative proteins sequences compared first to a database of known mycoplasma proteins and secondly to proteins from the UniProt/Swiss-Prot knowledgebase. Additionally, matches with HAMAP protein family permits to support homology annotation and raise the confidence level of annotation transfer. Other characterization features like functional domains constitute an additional support evidence of function assignment (Table 1). The results obtained from the various programs are parsed and stored in GFF3 format in a local MySQL database. The EMBL format is produced from the data stored in this database and deposited at the EBI EMBL database, the accession number is FM864216.

**Table 1:** Functional assignment criteria

Source	Known Protein	Putative	Hypothetical
Blastp (Mycoplasma DB)	Evalue < e-20	Evalue between	Evalue
		>e-20 and <e-4	> e-4 or No match
Blastp (Swissprot DB)	Evalue < e-20	Evalue between	Evalue
		>e-20 and <e-4	> e-4 or No match
HAMAP families	Confident protein family match	No confident protein family match	N/A
Interpro domains	If no HAMAP family match. Support evidence from at least one InterPro member database		Support evidence from one or none Interpro member database

Description of the criteria used to assign the genes products into the 3 following categories: Known Protein (known function: significant e-value and supported by confident protein family and functional domains), Putative protein (unclear function: twilight zone e-value but supported by functional domains) and Hypothetical protein (unknown function: non-significant e-value or no match in databases).

In order to assess the confidence of the results provided by our annotation pipeline, we used the known genome of *Mycoplasma hyopneumoniae *(strain 232) already annotated (NCBI entry AE017332) for comparing pipeline results with those provided at NCBI. The annotation pipeline identifies 741 CDS and 32 non-coding RNAs (ncRNAs) whereas only 691 CDS and 36 ncRNAS are known. The 91.1% of total genes were correctly predicted leaving only 23 genes not found by the predictor programs. Regarding the functional annotation, our pipeline provides 83.7% of correct gene annotations and even in some cases complements the existing one. 7.4% of CDS are wrongly annotated or in a different way. The 69 genes predicted in addition to known genes could be considered as false positives even if they also could represent new potential genes of *M. hyopneumoniae *genome (Table 2). Using results of *M. hyopneumoniae 232 *annotation, we evaluated the specificity and sensitivity of the pipeline and obtained a sensitivity of 92% and a specificity of 90% (Table 3).

**Table 2:** Results obtained for *M. hyopneumoniae *232 annotation

	All genes	CDS	ncRNA
Total of *M. hyopneumoniae *232 known genes	727	691	36
Total annotated genes using pipeline	773	741	32
Total correct predictions *	704	672	32
Total known genes not predicted *	23	19	4
Total correct gene annotations	647	618	29
Total predicted genes incorrectly or differently annotated	57	54	3
Total predicted genes in additionally annotated by pipeline	69	69	0

* CDS were predicted using Glimmer3 and ncRNAs were predicted using Blastn for 23S and 16S rRNAs, tRNAScan-SE for tRNAs and Infernal for other ncRNAs

**Table 3:** Evaluation of the sensitivity and the specificity of the pipeline based on re-annotation of the *M. hyopneumoniae *232 genome.

Total genes detected	FP	TP	FN	Specificity TP/(TP+FP)	Sensitivity TP/(TP+FN)
773	69	647	57	90%	92%

FP = False Positive, TP = True Positive, FN = False Negative

## Results

### General genome features

#### Composition and functional gene assignment, origin of replication

**Figure 1:**
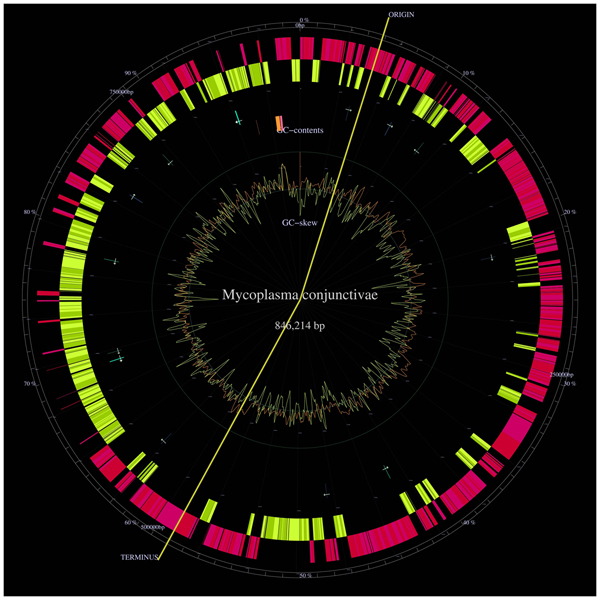
**Genome map**. *Mycoplasma conjunctivae *genome map generated by GenomeProjector tool and available in . This map represents from the outer ring in wards, genes on direct strand (pink), genes on complementary strand (yellow), tRNAs (green arrows), rRNAs (pink or orange stripes depending on the strand), GC content (brown lines), GC skew (yellow lines). The replication origin and terminus are predicted from the GC skew shift points and are in a different position than the one we found.

The genome consists of a single chromosome with a size estimated of about 0.9 Mbp. Currently, more than 95% of the genome is available as a single contig, but due to the presence of repeated sequences, we experienced difficulties in assembling the sequences to close the gap. The contig has a size of 846'214 bp with a G+C content of 29% (Figure 1).

A total of 734 genes have been computationally predicted. We found both 23S and 16S ribosomal RNAs in unique copies located next to each other. The 5S ribosomal RNA is located remotely of 23S and 16S genes. We identified 28 transfer RNAs covering all 20 amino acids. Other non-coding RNAs were found: bacterial RNase P class B, TPP riboswitch (THI element), tmRNA (proteolysis signal) and the bacterial signal recognition particle RNA.

Additionally, 699 genes were predicted as coding sequences for proteins. 49% of those genes have a clear homologue with a known function. 5.6% of those genes were annotated as "putative proteins" because the closest known protein was aligned with a marginal e-value. While the remaining 45% have unknown function, and were named as "hypothetical" proteins. From those hypothetical proteins, 75% matched non-significatively to other proteins and 25% are considered unique for *M. conjunctivae *because no match was obtained by blastp against both databases. A summary is shown in Table 4 and Table 5.

**Table 4:** Summary of *M. conjunctivae *genome features

Genome Features	
Length (bp)	846,214
G + C content (mole%)	29.0%

Functionally assigned protein CDSs	344
Putative protein CDSs	39
Hypothetical protein CDSs	316
23S rRNA	1
16S rRNA	1
5S rRNA	1
Bacterial RNase P class B	1
TPP riboswitch (THI element)	1
tmRNA	1
Bacterial signal recognition particle RNA	1
Transfer RNA genes	28

**Table 5:** Top 20 biological processes. Relevant information with a biological meaning was searched in priority. We list the top 20 of biological process that are accomplished by the newly annotated genes.

Biological process	Proteins matched
translation	68
metabolic process	32
transport	31
proteolysis	18
tRNA aminoacylation for protein translation	15
DNA repair	13
DNA replication	12
carbohydrate metabolic process	11
phosphoenolpyruvate-dependent sugar phosphotransferase system	11
regulation of transcription	10
DNA modification	9
glycolysis	9
ATP synthesis coupled proton transport	8
DNA methylation	7
DNA recombination	7
DNA integration	6
electron transport	6
biosynthetic process	5
nucleoside metabolic process	5
Protein folding	5

It is important to note that our method (homology based annotation) does not allow to distinguish between close homologues having different functions.

**Figure 2:**
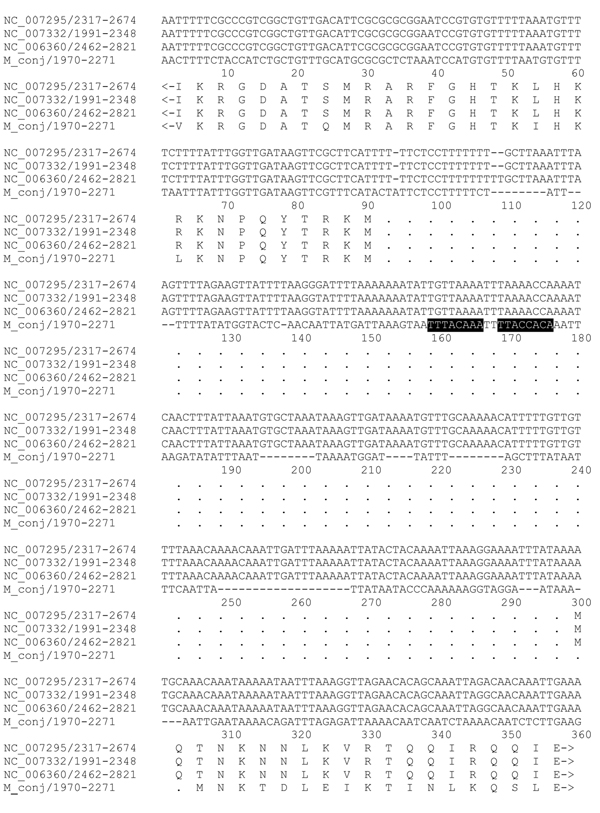
**Origin of replication**. The region between rpmH and dnaA genes for the 3 M. hyopneumoniae strains aligned with M. conjunctivae. The two putative dnaA boxes are shown in black. M. hyopneumoniae J (NC_007295), M. hyopneumoniae 7448 (NC_007332), M. hyopneumoniae 232 (NC_006360), M. conjunctivae (FM864216).

The origin of replication (oriC) was searched by comparing the sequences of 3 strains of *M. hyopneumoniae *with the sequence of *M. conjunctivae *in the region of *dnaA *gene (Figure 2). We attempted to identify several features that have been associated with replication origins in other bacterial species, including mollicutes. Bacterial origins of replication are typically located in the vicinity of the *dnaA *and *dnaN *genes. Usually several dnaA-box motifs are found within the intergenic regions around *dnaA *gene [26]. We searched unsuccessfully for the presence of consensus dnaA-box motifs with the pattern TTATC [CA]A [CA] using fuzznuc of the EMBOSS package [27]. When we used a slightly different, more relaxed dnaA-box consensus motifs TT [AT] [AC] [ACT]A [AC]A, two sequences matching each of these patterns were found between the *dnaA *and *rpmH *genes (Figure 2). However, well over 3,000 hits located throughout the rest of the genome were also seen. Therefore, the specificity of the pattern used to try to detect dnaA-box motifs was very low, decreasing our confidence in the significance of the sequences identified.

These findings are in contrast to the multiple dnaA-boxes found in the intergenic regions surrounding *dnaA *in other mollicutes [26]. In addition to the presence of dnaA-box motifs, replication origins can also frequently be identified by looking for biases in strand composition through measures such as the cumulative GC skew [28-30]. For *M. conjunctivae*, we found no significant asymmetries that can be readily detected with GC skew. The lack of a clear bias in *M. conjunctivae *is similar to that observed for the *M. hyopneumoniae *[31]. Therefore, the only significant feature of the *M. conjunctivae *genome that provides any possible indication of the location of the origin of replication is the presence of the *dnaA *gene. Otherwise, there are no features that allow definitive mapping of the origin to the intergenic region upstream of the *dnaA *gene, as seen in other bacteria.

### Potential pathogenic features

Bacteria have many ways to produce virulence that reside in the ability to adhere, invade and cause damage to host cells. Various strategies of pathogenicity such as cytolysins, toxins and invasins enable other bacteria to produce infection. In *Mycoplasma *species no such typical primary virulence genes have been found. Mycoplasmas seem rather to use intrinsic metabolic and catabolic functions to cause disease in the affected host and to ensure the microbe's survival. Our efforts to identify genes involved in the pathogenicity of *Mycoplasma conjunctivae *were concentrated on the one hand, try to find those primary virulence genes, toxins principally, rare in other mycoplasmas. On the other hand, on metabolic pathways that has been proposed by studies carried out in other mycoplasmas [8].

### Glycerol pathway

We found using manual blastp queries by an expert, the genes for a glycerol-3-phosphate dehydrogenase (*glpO*), a glycerol kinase (*glpK*), a glycerol uptake facilitator protein (*glpF*) and an ABC transporter system (Sn-glycerol-3-phosphate transport system permease) that are implicated in the glycerol metabolism producing cell damage, inflammation and disease in *Mycoplasma mycoides subsp. mycoides Small Colony *(SC) [8].

The pathway starts with the assimilation of glycerol by the ABC glycerol transporter (*gtsA*, *gtsB *and *gtsC*). Afterwards, the glycerol is phosphorylated into glycerol-3-phosphate, then oxidized by GlpO in presence of O_2 _into dihydroxyactone-phosphate (DHAP) producing one molecule of H_2_O_2_. H_2_O_2 _is released directly inside the host cells by the transmembrane GlpO protein leading to cell death [8]. The absence of any gene having a catalase or dismutase activity favors this hypothesis.

The identification of those genes in *Mycoplasma conjunctivae *constitutes an important discovery given that a relationship between the glycerol metabolism and cytotoxicity is established in the laboratory[8]. Further work to validate this hypothesis in *M. conjunctivae *is required and has been started in collaboration with a laboratory of the Institute for Veterinary Bacteriology (University of Bern).

### Toxins

Toxins constitute an important type of virulence factors in several bacteria. Thereby, we searched for toxins in *M. conjunctivae *and we found 3 proteins highly similar with toxins of *Treponema hyodysenteriae *(*Brachyspira hyodysenteriae*). Those proteins are Hemolysin A (*hlyA*), Hemolysin B (*hlyB*) and Hemolysin C (*hlyC*). The 3 genes are scattered on the genome.

Those proteins are present in other mycoplasmas, particularly *M. hyopneumoniae *and *M. capricolum*, and even if in those species, these toxins are not essential for pathogenicity mechanisms, it can not be excluded that these toxins contribute to the pathogenicity of *M. conjunctivae*.

### IS elements

Insertion sequences (IS) are short DNA elements that function as simple transposable elements by coding for proteins implicated in the transposition activity. Transposase and other regulatory protein are the proteins generally coded by IS elements: The transposase catalyses the enzymatic reaction allowing the IS to move. Regulatory proteins act by enhancing or inhibiting the transposition activity. The coding region in an insertion sequence is usually flanked by inverted repeats [32].

We found several genes coding for complete or partial transposases (Table 6). An IS*1138 *insertion element has particularly brought our attention. IS*1138 *elements belong to IS*3 *family are prevalent in other mycoplasmas and are the only (with IS*1138*b) that have been demonstrated directly to undergo autonomous transposition [32,33]. Interestingly a transposase for one IS*1138 *insertion elements is followed by homologues of a methylase *Hpa*I and a type II restriction enzyme HpaI from *Haemophilus parainfluenzae *forming a restriction-methylation cassette. The hypothesis of a horizontal transfer from *H. parainfluenza *to *M. conjunctivae *was formulated. We evaluated the G+C content of this cassette, but we did not observe a higher G+C content inside the cassette compared to the surrounding area. If the G+C content inside the cassette would be different from that of *M. conjunctivae *(29%) and similar to that of *H. parainfluenzae *(~41%) it could constitute an evidence of the transfer.

**Table 6:** List of *M. conjunctivae *transposases detected.

gene	Product	start	end	strand	length	Condition of the sequence
MCJ_00022	IS1138	23818	24999	-	1181	Complete
MCJ_00059	?	61624	61728	+	104	Partial
MCJ_00099	IS1138	97032	98213	+	1181	Complete
MCJ_00207	IS1138	192412	192660	-	248	Same transposase split in two ORFs
MCJ_00208	IS1138	192717	193088	-	371	
MCJ_00215	ISMag1	198177	199265	+	1088	Complete
MCJ_00399	IS1138	430830	432011	-	1181	Complete
MCJ_00428	IS1138	474034	474138	+	104	Partial
MCJ_00553	ISMHp1	639264	639500	-	236	Partial
MCJ_00635	IS1634AM	742041	743714	+	1673	Complete

### Comparative genomics

The list of proteins was classified and compared to 4 other mycoplasma genomes as shown in Table 7. The main difference with other mycoplasma is an apparently low carbohydrate and transport metabolism that could explain the need for a strong glycerol pathway, as well as the large number of hypothetical proteins probably due to the fully automatic annotation process.

**Table 7:** Genome comparison. Functional classification of proteins of 5 sequenced mycoplasma genomes

Functional categories	*Mycoplasma. hyopneumoniae 232*	*Mycoplasma pulmonis*	*Mycoplasma genitalium*	*Mycoplasma mobile*	***Mycoplasma conjunctivae***
	(892 kb)	(964 kb)	(816 kb)	(777 kb)	**(846 kb)**
Translation, ribosomal structure and biogenesis	100	108	108	108	**90**
Transcription	22	27	16	23	**16**
DNA replication, recombination and repair	75	118	49	72	**55**
Posttranslational modification, protein turnover	20	24	19	22	**15**
Energy production and conversion	25	29	20	29	**25**
Carbohydrate transport and metabolism	57	68	32	50	**30**
Amino acid transport and metabolism	25	27	17	24	**20**
Nucleotide transport and metabolism	19	22	20	18	**17**
Coenzyme transport and metabolism	9	12	12	17	**8**
Lipid transport and metabolism	6	10	9	9	**7**
Inorganic ion transport and metabolism	14	18	17	15	**5**
Other	118	124	87	107	**95**
No known function	201	195	78	139	**316**
Total CDSs	691	782	484	633	**699**

## Discussion

*Mycoplasma conjunctivae *is the fourteenth genome of a mycoplasma species that has been fully sequenced. Phylogenetically, the closest relative among the sequenced mycoplasmas is *M. hyopneumoniae *reflected by the high similarity of most of the proteins identified in *M. conjunctivae*.

The analysis of M. conjunctivae genome features, describes this organism as a typical mycoplasma, with a genome size and a G+C content within the range of other mycoplasma genomes. The comparison of mycoplasma genome sizes demonstrates that the sequence length is variable not only within the same genus but even among strains of the same species as shown in Table 8. Even if we do not know the final size of the genome, we expect a chromosome length of about 900'000 bp, size almost similar with the genome size of *M. hyopneumoniae*.

**Table 8:** Genome comparison. Genome size of 15 sequenced genomes of species belonging to Mycoplasma genus , including *M. conjunctivae*.

EMBL AC	Species name	Genome Size	G+C content
CU179680	*Mycoplasma agalactiae PG2 chromosome*	877,438	29.7%
CP001047	*Mycoplasma arthritidis 158L3-1*	820,453	30.7%
CP000123	*Mycoplasma capricolum subsp. capricolum ATCC 27343*	1,010,023	23.8%
AE015450	*Mycoplasma gallisepticum R*	996,422	31.5%
L43967	*Mycoplasma genitalium G37*	580,076	31.7%
AE017332	*Mycoplasma hyopneumoniae 232*	892,758	28.6%
AE017244	*Mycoplasma hyopneumoniae 7448*	920,079	28.5%
AE017243	*Mycoplasma hyopneumoniae J*	897,405	28.5%
AE017308	*Mycoplasma mobile 163 K*	777,079	25%
BX293980	*Mycoplasma mycoides subsp. mycoides SC str. PG1*	1,211,703	24%
BA000026	*Mycoplasma penetrans HF-2*	1,358,633	25.7%
U00089	*Mycoplasma pneumoniae M129*	816,394	40%
AL445566	*Mycoplasma pulmonis UAB CTIP*	963,879	26.6%
AE017245	*Mycoplasma synoviae 53*	799,476	28.5%
FM864216	***Mycoplasma conjunctivae***	**846,214**	**29%**

Globally the mycoplasma genomes have a characteristically low G+C content within the range of 23.8 to 40 mol% (Table 8). The highest G+C content found in *M. pneumoniae *and the lowest in *M. capricolum*. Regarding *Mycoplasma conjunctivae*, G+C content has a typical value of about 29%. The codon usage is similar to that of *M. hyopneumoniae *and opposite to that of *M. capricolum *and *M. mycoides *[31,34,35].

The presence of repeats across the genome was the principal difficulty for finishing the genome assembly. Insertion sequence (IS) elements are reported in the majority of mycoplasmas and in *M. conjunctivae *we found transposases for IS-elements in the genome. Some of those transposases genes are complete sequences and some other are fragmented showing a predicted length of less than 1000 bp. Since those insertion elements are nearly identical they created difficulties for assembling the genome.

The findings highlighted by this project, principally the glycerol pathway, require further experimental confirmation. In particular, the hypothesis for damaging the host cells by the glycerol metabolism need to be confirmed by demonstrating the localization of GlpO in the membrane and the release of H_2_O_2 _outside the cell. If this hypothesis can be verified, the possibility to block at any stage the glycerol pathway could constitute a candidate target for controlling the disease.

## Conclusion

In conclusion, we created an automatic pipeline to annotate a prokaryotic genome sequence using various tools for the prediction and the identification of the genes. This pipeline is customized for handling sequences of mycoplasma species.

We deposited the *Mycoplasma conjunctivae *genome fully annotated in the EMBL database (FM864216). Data stored into our local database can be searched and genome can be visualized through our website . Analysis of annotated genes gives new insights about potential mechanisms of pathogenicity as well as the possibility to go deeper into the knowledge of *Mycoplasma conjunctivae *and the IKC disease and opens the way to finding methods to prevent *M. conjunctivae *infections of domestic animals as reservoir for this pathogen and hence prevent IKC in wild animals.

## Competing interests

The authors declare that they have no competing interests.

## Authors' contributions

JF proposed and supported the project. SPCC created the pipeline and did the analysis. MAQ did the genomic library. TS and MAQ sequenced the clones. TS, GW and CW did the assembly. SPCC and LF wrote the manuscript.
